# A Network Pharmacology Study and Experimental Validation to Identify the Potential Mechanism of Heparan Sulfate on Alzheimer’s Disease-Related Neuroinflammation

**DOI:** 10.3390/biomedicines13010103

**Published:** 2025-01-05

**Authors:** Dong-Uk Kim, Bitna Kweon, Jinyoung Oh, Yebin Lim, Gyeongran Noh, Jihyun Yu, Hyang-Rin Kang, Tackmin Kwon, Kwang youll Lee, Gi-Sang Bae

**Affiliations:** 1 Department of Pharmacology, School of Korean Medicine, Wonkwang University, Iksan 54538, Republic of Korea; ckck202@naver.com (D.-U.K.); kbn306@naver.com (B.K.);; 2 Hanbang Cardio-Renal Syndrome Research Center, School of Korean Medicine, Wonkwang University, Iksan 54538, Republic of Korea; 3 Woori B&B Life Science Laboratory, Jeonju 54853, Republic of Korea; lynn@wooribnb.com (H.-R.K.);; 4 Research Center of Traditional Korean Medicine, Wonkwang University, Iksan 54538, Republic of Korea

**Keywords:** heparan sulfate, neuroinflammation, Alzheimer’s disease, network pharmacology, BV2

## Abstract

Background/Objectives: Heparan sulfate (HS) is a polysaccharide that is found on the surface of cells and has various biological functions in the body. Methods: The purpose of this study was to predict the pharmacological effects and molecular mechanisms of HS on Alzheimer’s disease (AD) and neuroinflammation (NI) through a network pharmacology analysis and to experimentally verify them. Results: We performed functional enrichment analysis of common genes between HS target genes and AD-NI gene sets and obtained items such as the “Cytokine-Mediated Signaling Pathway”, “Positive Regulation Of MAPK Cascade”, and “MAPK signaling pathway”. To confirm the predicted results, the anti-inflammatory effect of HS was investigated using lipopolysaccharide (LPS)-stimulated BV2 microglia cells. HS inhibited the production of nittic oxide, interleukin (IL)-6, and tumor necrosis factor-α in LPS-stimulated BV2 cells, but not IL-1β. In addition, HS inactivated P38 in the MAPK signaling pathway. Conclusions: These findings suggest the potential for HS to become a new treatment for AD and NI.

## 1. Introduction

Alzheimer’s disease (AD), the most common type of dementia, is a slowly progressive neurodegenerative disease that causes a decline in cognition, memory, and motor abilities and the loss of independence in daily life [1,2]. Currently, there are approximately 50 million people suffering from AD worldwide, and this number is expected to increase to 150 million by 2050. The progression of AD is very diverse and complex, and the pathogenesis is currently not clearly understood. The most influential hypothesis was that neuronal degeneration occurs due to abnormal aggregation of amyloid β or τ-protein in the cortex and limbic areas of the brain [3]. However, an AD treatment targeting these proteins was developed but failed repeatedly in the clinical stage [4]. Recently, neuroinflammation (NI), such as the activation of microglial cells and release of cytokines, has been attracting attention as a factor contributing to the pathological progression of AD [5].

Heparan sulfate (HS) is a polysaccharide with a variable structure that is found in the extracellular matrix or on the surface of the cell, usually associated with one of several types of core proteins [6]. HS that is bound to core proteins forms heparan sulfate proteoglycans (HSPGs) and exerts various physiological and pathologic functions [7]. In particular, HS is involved in the pathological progression of AD by regulating the clearance of amyloid beta and amyloid beta-induced neurotoxicity in the brain [8,9]. Although the anti-AD effects of HS have been elucidated, studies of NI in HS are still scarce.

Network pharmacology is widely employed to investigate the interactions between drugs and various targets [10]. Recently, network pharmacology, a systems biology-based method, was evaluated as a cost-effective and logical approach to drug development [11]. In addition, in complex diseases such as AD, where the etiology is not clearly understood, treatment considering multiple targets may be more effective than treatment targeting a single target [12,13]. Therefore, a network pharmacology analysis was performed to determine therapeutic targets and signaling pathways that are closely related to AD and NI. In addition, the anti-neuroinflammatory effect of HS was evaluated in the mouse microglial cell line BV2.

## 2. Results

### 2.1. Network Pharmacology Analysis with HS and AD-NI

**Figure 1:**
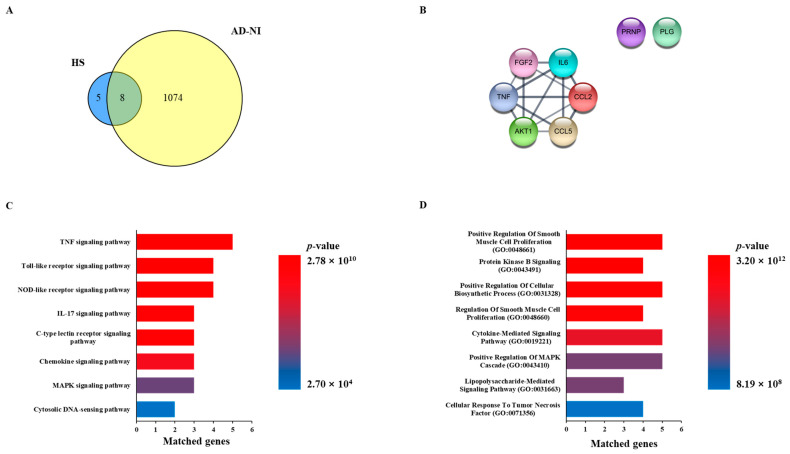
Network pharmacology analysis of Heparan sulfate (HS) with Alzheimer’s disease (AD) and neuroinflammation (NI). (**A**) Venn diagram of related targets between HS and gene sets of AD-NI. (**B**) Common genes of HS and AD-NI. (**C**) Enrichment analysis based on KEGG pathway of common genes. (**D**) Enrichment analysis based on GO biological process of common genes.

A total of 13 genes related to HS were extracted from the Pubchem database, and the HS network was constructed. We searched the disease database Genecards with the keywords “Alzheimer’s disease” and “Neuroinflammation” to obtain a list of genes associated with each disease. In order to select AD and NI genes, common genes were extracted from the disease-related gene list, which was called the AD-NI gene set. As a result, eight genes overlapped between the HS network and the AD-NI gene set, as follows: AKT1, CCL2, CCL5, FGF2, PLG, PRNP, IL6, and TNF (Figure 1A,B).

### 2.2. Functional Enrichment Analysis with HS and AD-NI

In order to investigate the function of the HS-related genes and predict the mechanism of action for AD-NI, KEGG pathway and GO enrichment analyses were performed. As a result of the KEGG pathway analysis, 137 potential mechanisms involving eight common genes were obtained. Among them, the top eight items, “TNF signaling pathway,” “Toll-like receptor signaling pathway”, “NOD-like receptor signaling pathway”, ”IL-17 signaling pathway”, “C-type lectin receptor signaling pathway”, “Chemokine sissgnaling pathway”, “MAPK signaling pathway”, and “Cytosolic DNA-sensing pathway”, were selected based on the *p*-value (Figure 1C). In addition, we obtained 691 items related to eight common genes in the GO biological process analysis and obtained the top eight items of “Positive Regulation Of Smooth Muscle Cell Proliferation (GO:0048661)”, “Protein Kinase B Signaling (GO:0043491)”, “Positive Regulation Of Cellular Biosynthetic Process (GO:0031328)”, “Regulation Of Smooth Muscle Cell Proliferation (GO:0048660)”, “Cytokine-Mediated Signaling Pathway (GO:0019221)”, “Positive Regulation Of MAPK Cascade (GO:0043410)”, “Lipopolysaccharide-Mediated Signaling Pathway (GO:0031663)”, and “Cellular Response To Tumor Necrosis Factor (GO:0071356)” based on the *p*-value (Figure 1D).

### 2.3. Influence of HS on BV2 Cell Viability

**Figure 2:**
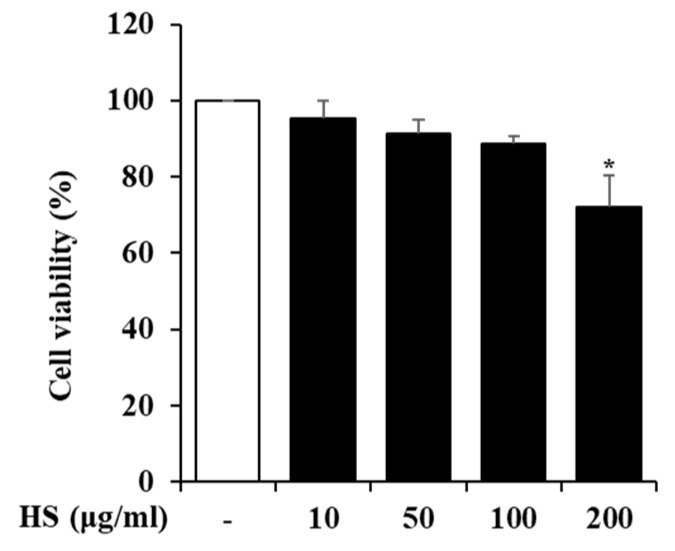
The cytotoxicity of the HS in BV2 cells. The BV2 cells were cultured with various concentrations of HS for 24 h. The cell viability was measured by MTT assay. Data are presented as means ± standard error of the mean (SEM). * *p* < 0.05 vs. saline treatment alone.

To determine the maximum dose of HS to be treated in BV2 cells, the cell viability was measured using a 3-(4,5-Dimethylthiazol-2-yl)-2,5 Diphenyl Tetrazolium Bromide (MTT) reagent. BV2 cells were treated with 10, 50, 100, or 200 μg/mL HS, and their cell viability was measured 24 h later. As shown in Figure 2, there was no cytotoxicity up to HS 100 μg/mL, and subsequent experiments were conducted with the maximum dose set at 100 μg/mL based on the MTT analysis results.

### 2.4. Effect of HS on Nitric Oxide (NO) Production in LPS-Stimulated BV2 Cells

**Figure 3:**
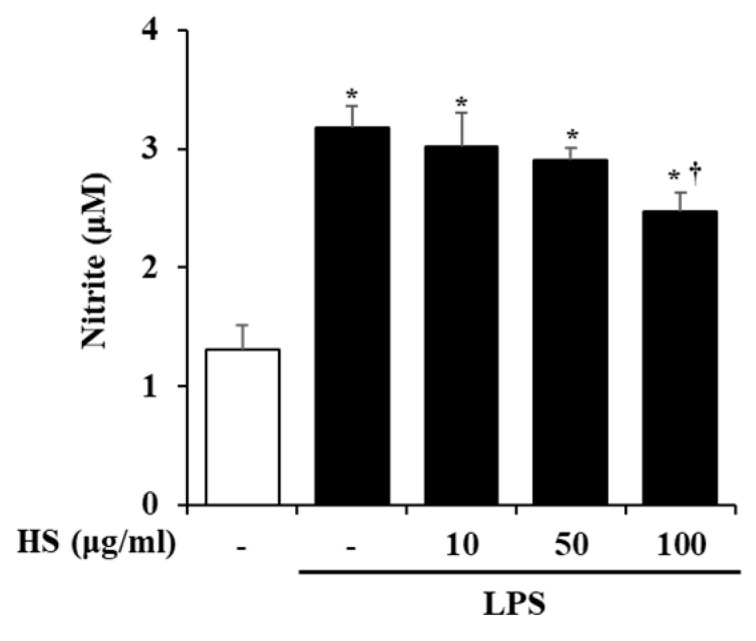
Effect of HS on nitric oxide (NO) production in lipopolysaccharide (LPS)-stimulated BV2 cells. BV2 cells were pretreated with HS (10, 50, and 100 μg/mL) for 1 h and co-incubated with LPS (1 μg/mL) for 24 h. NO in supernatant was measured by Griess assay. Data are presented as means ± SEM. * *p* < 0.05 vs. saline treatment alone; † *p* < 0.05 vs. LPS treatment alone.

The activation of BV2 cells by LPS stimulation was evaluated based on the level of NO production. BV2 cells were pretreated with HS 10, 50, or 100 μg/mL for 1 h and then treated with LPS 1 μg/mL for 24 h. Compared to the control group, the production of NO was significantly increased in the LPS-treated group. However, the NO production that was increased by LPS treatment in the HS-treated group was decreased in a dose-dependent manner (Figure 3).

### 2.5. Effect of HS on Pro-Inflammatory Factor Expression in LPS-Stimulated BV2 Cells

**Figure 4:**
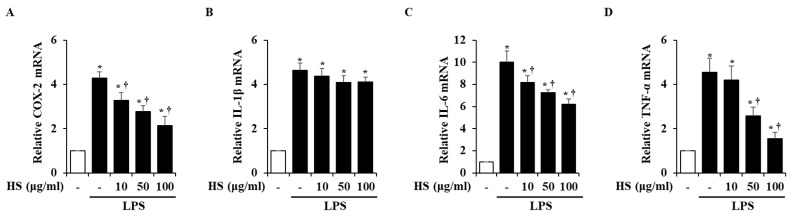
Effect of HS on expression of pro-inflammatory factors in LPS-stimulated BV2 cells. BV2 cells were pretreated for 1 h with 10, 50, and 100 μg/mL of HS and co-incubated with 1 μg/mL of LPS for 6 h. mRNA levels of (**A**) cyclooxygenase (COX)-2, (**B**) interleukin (IL)-1β, (**C**) IL-6, and (**D**) tumor necrosis factor (TNF)-α were evaluated by using real-time RT-PCR. Data are presented as means ± SEM. * *p* < 0.05 vs. saline treatment alone; † *p* < 0.05 vs. LPS treatment alone.

To investigate the mRNA expression levels of inflammatory factors, real-time reverse transcription polymerase chain reaction (RT-PCR) was conducted. Inflammatory factors, particularly cyclooxygenase (COX)-2, interleukin (IL)-1β, IL-6, and tumor necrosis factor (TNF)-α, are known to induce inflammatory responses [14,15]. In the LPS treatment group, the mRNA levels of the inflammatory factors were significantly increased compared to the control group. However, when treated with 10, 50, or 100 μg/mL of HS, the COX-2, IL-6, and TNF-α mRNA levels were dose-dependently suppressed, but the expression of IL-1β mRNA was not suppressed (Figure 4).

### 2.6. Effect of HS on Mitogen-Activated Protein Kinase (MAPK) Signaling Activation in LPS-Stimulated BV2 Cells

**Figure 5:**
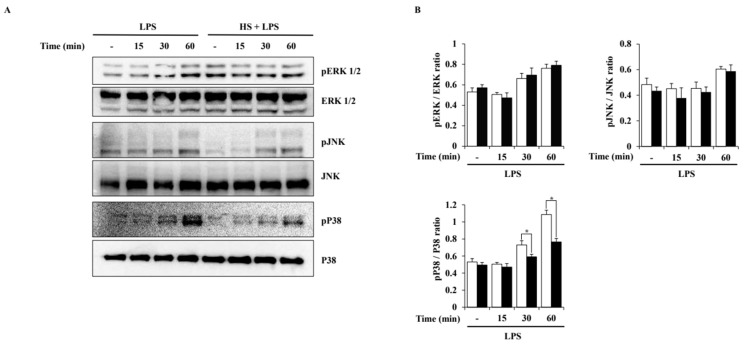
Effect of HS on MAPK activation in LPS-stimulated BV2 cells. BV2 cells were pretreated with 100 μg/mL of HS for 1 h and stimulated with LPS (1 μg/mL) for 0, 15, 30, and 60 min. (**A**) MAPK phosphorylation was evaluated through Western blotting. (**B**) Relative density ratio of Western blotting. Data are presented as means ± SEM. * *p* < 0.05 vs. LPS treatment alone.

To evaluate the activation of MAPK pathways induced by LPS in BV2 cells, a Western blot analysis was performed. Phosphorylation of extracellular signal-regulated kinase (ERK) 1/2, c-Jun N-terminal kinase (JNK), and P38 was observed at 15, 30, or 60 min after LPS treatment. HS treatment at 100 μg/mL only suppressed the phosphorylation of P38 and did not affect the phosphorylation of ERK1/2 and JNK (Figure 5).

## 3. Discussion

Despite extensive research in a variety of fields over a long period of time, no effective treatment for AD has yet been developed, causing suffering for many patients and their families [16]. Recently, neuroinflammation has emerged as a cause of neurodegenerative diseases such as AD, and research relating to this is being actively conducted [17,18,19]. Therefore, this study predicted potential targets and pathways of NI associated with AD that may be affected by HS through network pharmacology and experimentally verified them.

A network pharmacology analysis was performed to investigate the correlation between HS and AD-NI, and eight common genes, namely, AKT1, CCL2, CCL5, FGF2, IL6, PRNP, PLG, and TNF, were sorted (Figure 1A). The common genes formed a network with eight nodes and fourteen edges, and their potential biological processes and mechanisms, including the “Cytokine-Mediated Signaling Pathway (GO:0019221)”, “Positive Regulation of MAPK Cascade (GO:0043410)”, and “MAPK signaling pathway”, were investigated through functional enrichment analysis using KEGG pathways and GO biological processes (Figure 1B–D). This network study revealed that IL6 and TNF affect many biological processes and mechanisms related to AD-NI, and many other studies also suggest that they play important roles in AD and NI [20,21,22]. In summary, these results suggested that HS may affect AD and NI through the regulation of cytokine and MAPK signaling.

In this study, we validated the network pharmacology analysis using BV2, a microglial cell line, which is a type of macrophage that is present in the central nervous system (CNS). Microglia are thought to be involved in the development and maturation of the CNS and participate in developmental synaptic pruning, maintenance of synaptic plasticity, immune responses, and neuronal apoptosis [23,24]. When pathological damage occurs in the CNS, microglial receptors can recognize abnormal proteins, pathogens, and cellular debris and activate microglia [25]. Activated microglia degrade them through phagocytosis and increase the expression of inflammatory mediators and cytokines, which are components of the neuroinflammatory process [26,27]. However, aged microglia are dysfunctional and persistently activated, which can adversely affect normal neurons and lead to the development of neurodegenerative diseases like AD [28]. Therefore, research on NI associated with AD should also be conducted. However, although research on the anti-Alzheimer’s effect of HS regulating amyloid beta has been conducted, research on neuroinflammation is still scarce [8,9]. NO is a substance that mainly regulates physiological functions in the cardiovascular and nervous systems and is also known to be involved in inflammatory responses [29,30]. In particular, microglial cells in the brain produce NO to respond to inflammation, but excessive levels of NO worsen neuroinflammation and cause neuronal death [31,32]. In this study, we found that HS regulates neuroinflammation by suppressing excessive NO production in BV2 cells (Figure 3).

Next, changes in factors involved in the inflammatory process, such as COX-2, IL-1β, IL-6, and TNF-α, were observed at the mRNA level. The COX-2 expression in the brain is known to be associated with inflammatory activity and is thought to be an important mediator in the process of neuroinflammation leading to neurodegenerative diseases [33]. Activated microglia produce inflammatory cytokines to protect the CNS through innate defense mechanisms [34,35]. However, excessively secreted cytokines can cause immune activation and cytotoxicity, which can lead to neurodegeneration and neuronal death [36]. In this study, LPS-stimulated BV2 cells showed increased expression of inflammatory factors such as COX-2, IL-1β, IL-6, and TNF-α at the mRNA level. However, HS treatment decreased the expression of COX-2, IL-6, and TNF-α (Figure 4). This finding is consistent with the previous network analysis, suggesting that HS may regulate IL-6 and TNF-α.

HS was confirmed to have the potential to regulate the MAPK signaling pathway through functional enrichment analysis. Therefore, in this study, the effect of HS on the MAPK and NF-κB signaling pathways in LPS-stimulated BV2 cells was evaluated through Western blotting. In this study, LPS treatment activated P38, ERK1/2, and JNK in BV2 cells, but HS significantly inactivated P38 (Figure 5). MAPK P38 is also called stress-activated protein kinase, because it is mainly activated by cytokines and extracellular stresses, and has been extensively studied in inflammatory diseases [37]. Other studies have shown that P38 is a key regulator of microglial activation and inflammatory responses and is particularly involved in amyloid beta-induced neurotoxicity [37,38,39]. Therefore, modulating P38 may be a therapeutic target for NI and any associated AD.

The purpose of this study was to predict the targets and mechanisms of HS for AD and NI through network pharmacology analysis and to experimentally validate them. Through a functional enrichment analysis of common genes between HS and AD-NI, we predicted that cytokines and MAPK signaling would be the mechanism of action of HS. Corresponding to these predictions, HS suppressed the expression of IL-6 and TNF-α and inactivated P38 MAPK signaling in LPS-stimulated BV2 cells. In conclusion, HS may be one of the key candidates for NI and AD by inhibiting IL-6, TNF-α, and P38 MAPK signaling. This study is believed to provide important clues for the development of AD therapeutics through NI.

## 4. Materials and Methods

### 4.1. Network Construction of Common Genes Between HS and AD-NI-Targeted Genes

To construct the HS network, HS-related genes were obtained from the PubChem database (https://pubchem.ncbi.nlm.nih.gov/, accessed on 20 November 2024). A total of 100 genes that co-occurred with HS in the literature were collected from PubChem. As a result, a total of 13 genes were obtained by verifying whether the actual literature contained information about HS and its genes. And the confidence score for network construction of these genes in the String database (http://www.string-db.org/, accessed on 25 November 2024) was set to 0.7. Gene sets of diseases were collected by searching for “Alzheimer’s disease” and “Neuroinflammation” as keywords in GeneCards (http://www.genecards.org/, accessed on 27 November 2024), a disease database. A total of 13 genes in the HS Network were compared with the 1082 genes in the gene set of AD-NI to assess any correlation between HS and AD-NI. Common genes in HS and AD-NI were used to construct a network to investigate related pathways.

### 4.2. Functional Enrichment Analysis

To predict potential molecular functions and molecular interactions for AD-NI in common genes, a functional enrichment analysis was conducted by identifying terms in the KEGG pathways and GO processes using the Enrichr database (https://maayanlab.cloud/Enrichr/, accessed on 27 November 2024). The KEGG pathways and GO processes were sorted by their *p*-value to obtain the top 8 items.

### 4.3. BV2 Cell Culture

BV2 microglia cells were purchased from AcceGen (Fairfield, NJ, USA). The cells were grown in Roswell Park Memorial Institute (RPMI) 1640 and supplemented with 10% fetal bovine serum (FBS) and 1% penicillin/streptomycin. They were cultured in an environment at 37 °C with 5% CO_2_.

### 4.4. MTT Assay

To investigate the effect of HS on BV2 cells, their cell viability was evaluated by the MTT assay. The BV2 cells were seeded at 2 × 10^5^ cells/well in a 24-well plate and treated with various concentrations of HS (10, 50, 100, and 200 μg/mL). After 24 h, the cells were treated with an additional 5 mg/mL of MTT reagent and incubated for 30 min. Then, the supernatant was removed, the formazan was dissolved in dimethyl sulfoxide, and the optical density was measured using SpectraMax M2 (Molecular Devices; San Jose, CA, USA) at 540 nm.

### 4.5. Measurement of NO

The BV2 cells were incubated in a 24-well plate at 2 × 10^5^ cells/well. The cells were pretreated with HS 10, 50, and 100 μg/mL. After 1 h, the BV2 cells were treated with lipopolysaccharide (LPS) at 1 μg/mL and stimulated for 24 h. The supernatant was reacted with Griess reagent at the same ratio and measured using SpectraMax M2 at 540 nm.

### 4.6. Real-Time RT-PCR

The BV2 cells were incubated at 1 × 10^6^ cells/well in 6-well plates. Various concentrations of HS (10, 50, and 100 μg/mL) were pretreated on BV2 cells for 1 h. Then, the BV2 cells were treated with LPS (1 μg/mL) and incubated for 6 h. To obtain total RNA from the cells, the Easy-Blue™ RNA Extraction Kit (cat. no. 17061; iNtRON Biotechnology, Inc., Sung-nam, Republic of Korea) was used according to the manufacturer’s instructions. And then, the extracted RNA was reverse-transcribed into cDNA by the ReverTra AceTM qPCR RT Kit (TOYOBO, Osaka, Japan) following the manufacturer’s instructions. The target mRNA was amplified with the SYBR Premix kit (Applied Biosystems; Waltham, MA, USA) under the following thermocycling conditions: 10 min at 95°C for one cycle, followed by 40 cycles of 15 s at 95°C, 30 s at 60°C, and 1 min at 72°C. The primer sequences used in the experiment are shown in Table 1.

### 4.7. Western Blotting Assay

The BV2 cells were incubated at 5 × 10^6^ cells/dish in 6 cm dishes. The BV2 cells were divided into two groups: treated with or without HS 100 μg/mL. After 1 h, the BV2 cells were treated with LPS 1 μg/mL for 0, 15, 30, and 60 min, the supernatant was removed, and the cells were lysed using a RIPA lysis buffer (iNtRON biotechnology, Sungnam, Republic of Korea) containing 1% Xpert Duo inhibitor cocktail (P3300-005; GenDEPOT, LLC., Baker, TX, USA). Then, the protein samples were resolved by a sodium dodecyl sulfate (SDS)-polyacrylamide gel and transferred electrophoretically to a membrane. The membranes were blocked with 5% skim milk for 2 h at room temperature (RT) and then incubated with primary antibodies (1:1000) overnight at 4 °C. The list of primary antibodies used in the experiments is as follows: phospho-P38 (pP38) (#9211); P38 (#9212); pJNK (#9251); JNK (#9252); pERK 1/2 (#9101); ERK1/2 (#9102); Iκ-Bα (#9242); and GAPDH (#2118). All primary antibodies were purchased from Cell Signaling Technology (Danvers, MA, USA). P38, JNK, ERK 1/2, and GAPDH were used as loading controls. After washing the membrane, Goat anti-rabbit IgG (H + L)–horseradish peroxidase (HRP) (SA002-500; GenDEPOT, LLC., Baker, TX, USA) was used as a secondary antibody and incubated at RT for 1 h. The proteins were visualized using an enhanced chemiluminescence detection system (Amersham; Buckinghamshire, UK).

### 4.8. Statistical Analysis

All data are presented as the mean ± standard error of the mean (SEM). Statistical analyses were conducted using one-way analysis of variance with the SPSS statistics 29.0 (SPSS Inc., Chicago, IL, USA). Differences between experimental groups were determined by post hoc analysis using Duncan’s method. A *p*-value < 0.05 considered statistically significant.

## Figures and Tables

**Table 1:** Sequences of primers used for real-time polymerase chain reaction (PCR).

Gene	Primer	
*COX-2*	F (5′−3′)	GGTGGCTGTTTTGGTAGGCTG
	R (5’−3′)	GGGTTGCTGGGGGAAGAAATG
*IL-1β*	F (5′−3′)	CCTCGTGCTGTCGGACCCAT
	R (5′−3′)	CAGGCTTGTGCTCTGCTTGTGA
*IL-6*	F (5′−3′)	CCGGAGAGGAGACTTCACAG
	R (5′−3′)	CAGAATTGCCATTGCACAAC
*TNF-α*	F (5′−3′)	AACTAGTGGTGCCAGCCGAT
	R (5′−3′)	CTTCACAGAGCAATGACTCC
*GAPDH*	F (5′−3′)	TGTGTCCGTCGTGGATCTGA
	R (5′−3′)	TTGCTGTTGAAGTCGCAGGAG

## Data Availability

The dataset is available on request from the authors.
